# Skeletal maturity and oxygen uptake in youth soccer controlling for concurrent size descriptors

**DOI:** 10.1371/journal.pone.0205976

**Published:** 2018-10-18

**Authors:** Anderson S. Teixeira, Luiz G. A. Guglielmo, Juliano Fernandes-da-Silva, Jan M. Konarski, Daniela Costa, João P. Duarte, Jorge Conde, João Valente-dos-Santos, Manuel J. Coelho-e-Silva, Robert M. Malina

**Affiliations:** 1 Physical Effort Laboratory, Sports Center, Federal University of Santa Catarina, Florianópolis, Brazil; 2 Research Group for Development of Football and Futsal, Sports Center, Federal University of Santa Catarina, Florianópolis, Brazil; 3 Poznań University of Physical Education, Theory of the Sport Department, Poznań, Poland; 4 CIDAF (uid/dtp/04213/2016), University of Coimbra, Coimbra, Portugal; 5 Faculty of Sport Sciences and Physical Education, University of Coimbra, Coimbra, Portugal; 6 Portuguese Foundation for Science and Technology (SFRH/BD/101083/2014), Lisbon, Portugal; 7 School of Health and Technology, Polytechnic Institute of Coimbra, Coimbra, Portugal; 8 Portuguese Foundation for Science and Technology (SFRH/BPD/100470/2014), Lisbon, Portugal; 9 Institute for Biomedical Imaging and Life Sciences (IBILI), Faculty of Medicine, University of Coimbra, Coimbra, Portugal; 10 Faculty of Physical Education and Sport, Lusófona University of Humanities and Technologies, Lisbon, Portugal; 11 Department of Kinesiology and Health Education, University of Texas, Austin, Texas, United States of America; Nanyang Technological University, SINGAPORE

## Abstract

Interrelationships among skeletal maturity status, body size, ventilator thresholds (VT) and peak oxygen uptake (*V*O_2peak_) were considered in 47 adolescent male soccer players aged 12.5–15.4 years. Body mass, stature, and the triceps and subscapular skinfolds were measured. The latter were used to estimate fat mass and fat-free mass. Skeletal age was assessed with the Fels method. *V*O_2peak_ and *V*O_2_ at the first (VT_1_) and second (VT_2_) ventilatory thresholds were determined during an incremental maximal exercise test on a motorized treadmill. Ratio standards and allometric models were used in the analysis. Scaling exponents suggested linearity for all combinations between size descriptors and physiological variables, except between log-transformed values of VT_1_ and body mass (mL·kg^-0.801^·min, 95%CI: 0.649 to 0.952). Early maturing players attained greater values than players classified as “on-time” in skeletal maturity for the three ventilatory parameters expressed in absolute terms (d ranged from 0.65 to 0.71). The differences were attenuated after normalizing for mass descriptors using ratio standards and scaled variables (d ranged from 0.00 to 0.31). The results suggested significant variability between maturity groups when moving from VT_1_ to maximal metabolic conditions expressed by unit of stature (VT_1_: t = -2.413, p = 0.02, d = 0.60; VT_2_: t = -2.488, p = 0.02, d = 0.65; *V*O_2peak_: t = -2.475, p = 0.02, d = 0.65). Skeletal maturity status and associated variation in overall body size affects VT_1_, VT_2_ and *V*O_2peak_. The observed scaling of ventilatory outputs for body size may be related to the better running economy and smaller body size of average maturing athletes.

## Introduction

Assessment of the fitness level of soccer players along with information about match performance are essential components of youth soccer particularly for the purpose of long-term athletic training [1]. Available evidence suggests that adult male players covered approximately 10–12 km during a soccer match [2]. Although less abundant, studies of youth soccer report shorter distances covered during competitions [3]: 6.5 km (Under-13), 7.4 km (Under-14), 8.1 km (Under-15). Seven international matches were analysed during the Under-20 South American Championship using global positioning technology and noted that in the final 15 minutes of a game, total distance and high-intensity running were 20–35% lower than during the initial 15 minutes [4]. More recently, the influence of predicted biological maturity status on match performance based on global positioning system in elite youth male soccer players 8–16 years was examined in 80 outfield players in a British academy [5]. Players of contrasting maturity status differed in total distance covered during a match; late maturing players covered greater distance per hour while early maturing spent a longer percentage of time in high speed running [5]. Though of interest, the latter results should be considered in the context of the limitations of the protocol used to predict maturity status [6].

Activities of young players during competitions were generally sustained at about 85% of individual peak heart rate and 75–80% of peak oxygen uptake (*V*O_2peak_) [7, 8]. Corresponding match performance data of youth players are rather limited. Although significant correlations between time-motion variables and *V*O_2peak_ were not noted among adolescent soccer players [9], it is well recognized that a better aerobic fitness level is associated with high external training/match loads [3] and an improved tolerance to fatigue in high-intensity activities [10], suggesting a better ability to maintain physical performance for a prolonged period. Not surprisingly, ventilatory thresholds (VT) and running speed were suggested for monitoring aerobic training in soccer players, specifically during the early phase of the preseason [11]. Anthropometric characteristics, physical fitness and technical skills of adolescent soccer players were compared by competitive level and playing position (goalkeeper, central defender, fullback, midfield, forward) and it was found that stature and body mass discriminated elite from non-elite players among goalkeepers and central defenders [12]. By inference, coaches would benefit from better awareness of relationships among skeletal maturity status, body size and ventilatory parameters for the purpose of talent development and conditioning of youth soccer players.

Inter-individual variability in absolute *V*O_2peak_ was strongly correlated with body size, but was also influenced by function of the lungs, heart and skeletal muscle [13]. Body size indicated as body mass, stature, fat-free mass and thigh volume have been identified as significant predictors of aerobic fitness in adolescents [14–17]. Oxygen uptake is often expressed per unit of body mass (ratio standard: ml∙min^-1^∙kg^-1^), although this has theoretical and mathematical limitations [18]. Allometric models were suggested as an valid alternative approach to normalize physiological variables for inter-individual variability in body size [18]. Biological maturity status introduces further inter-individual variability in body size descriptors. Consequently, inadequate normalization for body size may contribute to misleading in the interpretation of ventilatory oxygen parameters during an incremental maximal test for soccer players contrasting in biological maturity and body size. Skeletal age (SA) of the hand-wrist is consensually considered the best of biological maturity status [19] and available literature combining maturation and *V*O_2peak_ in adolescent athletes has not systematically used this maturity indicator. Actually, the relationships between skeletal maturation and *V*O_2peak_ and VT in youth sports have not been extensively addressed, particularly in soccer [14–16, 20]. In this context, the purpose of the present study is to evaluate the interrelationships among skeletal maturity status, body size as reflected in body mass, fat-free mass and stature, and *V*O_2_ outputs in adolescent male soccer players 12 to 15 years of age. It was hypothesized that ventilatory oxygen parameters would be substantially explained by inter-individual variance in whole-body size descriptors that are influenced by skeletal maturation. By inference, it was also hypothesized that differences on *V*O_2_ outputs between soccer players of contrasting skeletal maturity status would be attenuated after adequate normalization for body size descriptors.

## Materials and methods

### Research design and procedures

This cross-sectional study describes interrelationships among ventilatory oxygen uptake, body size descriptors and skeletal maturity status and includes a comparison between adolescent soccer players of contrasting skeletal maturity status. The *Ethics Committee* from the Federal University of Santa Catarina (protocol 2004-Federal University of Santa Catarina/2011) approved the research proposal. It was conducted according to the standards established by the declaration of Helsinki.

Adolescent soccer players were recruited in several soccer academies of professional clubs competing at the Brazilian national level after obtaining permission from the respective managers. Parents or legal guardians were informed about the nature of the study including objectives, protocols and related risks, and provided informed written consent. Participation was voluntary and players provided assent after being informed that they could withdraw from the study at any time.

All measurements were completed at the *Federal University of Santa Catarina* during the competitive season at the same time of day (afternoon, usually 16:00–19:00h). Participants were instructed to avoid heavy training for 48 h before the testing session. They were also instructed to avoid caffeinated drinks. The players followed a standardized diet provided at the soccer club academy including the day before testing (~50–60%, 30–25% and 20–15% of total energy intake composed of carbohydrates, fat, and protein, respectively). Anthropometry and the maximal running test were conducted in the laboratory on a single occasion. Air temperature and humidity were kept constant throughout the incremental treadmill test (20–22°C, 50–60% humidity). After the visit to the laboratory, players were then transported within the week to the Santa Catarina University Hospital for a radiograph of the left hand-wrist for the purpose of assessing SA.

### Participants

The sample included 47 male soccer players aged 12.4–15.4 years (S1 File). Inclusion criteria were: (*i*) adolescent male soccer players; (*ii*) chronological age between 12 and 16 years; (*iii*) absence of injuries and clinical signs of cardiovascular or pulmonary conditions which would affect the achievement of maximum performances during an incremental test; (*iv*) a minimum of three years of experience in competitive soccer; (*iv*) outfield players (goalkeepers were not included since they are typically selected for larger body size, specifically stature, and are not expected to cover long distances during a match). Players were classified as Under-14 (12.0 to 13.9 years) and Under-16 (14.0 to 15.9 years) according to the Soccer Federation of Santa Catarina (associated with the Brazilian Soccer Confederation). The players trained 3–5 regular sessions of 90–120 minutes per week and usually participated in one official game (usually on Saturdays). Of interest, three of the Under-16 players were selected for the Brazilian national team. Chronological age (CA) was calculated as the difference between date of birth and date of the hand-wrist radiograph. Goalkeepers were excluded.

### Anthropometry

Measurements were taken by a single, experienced individual following standard procedures [21]. Body mass was measured to the nearest 0.1 kg (Scale Soehnle, Murrhardt, Germany). Stature was measured to the nearest 0.1 cm (Stadiometer Sanny, American Medical do Brazil, Brazil). The triceps and subscapular skinfolds were measured to 0.1 mm with a skinfold caliper (Adipometer, Cescorf, Porto Alegre, Brazil). Intra-observer technical errors of measurement were 0.2 cm for stature, 0.3 mm for the triceps and 0.3 mm for the subscapular skinfolds. Stage of pubic hair, using the criteria of Tanner [22], was assessed in a separate room by an experienced paediatrician who was a professor in the local medical school. The prediction equation for percentage fat mass which uses the triceps and subscapular skinfolds is specific for pubertal status [23]:

$$\text{Fat mass}\;\left(\text{\%}\right)=1.21\;\times \;\left(\text{X}\right)-0.008\;\times \;{\left(\text{X}\right)}^{2}+\;\text{constant}$$ (1)

where X is the sum of the triceps and subscapular skinfolds (mm) and the constant (intercept) is specific for pubertal status: 1.7 for pre-pubescent, 3.4 for pubescent and 5.5 for post-pubescent males.

### Skeletal maturation

The SA was estimated with the Fels method [24], which utilizes specific criteria for each bone of the hand-wrist and the ratios of linear measurements of epiphyseal and metaphyseal widths. Grades for each indicator and width measurements were entered into the Fels program (Felshw 1.0 Software) to estimate SA and the associated standard error of estimate. Details of the method and a critical overview of its applications were presented elsewhere [19]. All radiographs were assessed by a single, experienced observer. Independent assessments of 14 radiographs by the observer and an experienced assessor were used to examine quality control of the assessments. The two assessors performed 391 observations with an agreement rate of 93.1% and, as previously reported [25], intra-individual difference (-0.12 ± 0.34), inter-observer error of measurement (0.25 years) and coefficient of variance (CV% = 1.41) were within normal range. Players were classified into contrasting maturity groups based on the difference of SA minus CA. An SA within ± 1.0 year of CA was classified as average or on time. An SA in advance of CA by >1.0 year was classified as early, while an SA delayed relative to CA by > -1.0 year was classified as late [19]. Among the 47 participants, 29 were classified as early maturing (62%) and 18 were average (38%); no players had an SA that classified them as late maturing and no players were skeletally mature.

### Incremental maximal treadmill test

Oxygen uptake outputs (maximal and ventilatory thresholds) were obtained during an incremental running test on a motorized treadmill (Ibramed Millenium Super, Brazil). Pulmonary gas exchange was measured breath-by-breath using an automated open-circuit gas analysis system (Quark PFTergo, Cosmed, Rome, Italy). The gas analysers were calibrated immediately before each test using ambient air (assumed to contain 20.94% oxygen and 0.03% carbon dioxide), and certified alpha standard gases containing 16.0% oxygen and 5.0% carbon dioxide (White Martins Ltda, Osasco, Brazil). The turbine flowmeter used for the determination of minute ventilation was calibrated with a 3-L syringe (Quark PFT Ergo, Cosmed, Rome, Italy). Gas exchange responses were interpolated to 1-s intervals and averaged every 15s. After a 3-minute warm-up running at 6.6 km·h^-1^, the test started at 7.2 km·h^-1^ with subsequent increments of 0.6 km·h^-1^ every minute until voluntary exhaustion [26]. The slope was kept constant at 1% across the entire test. All players were verbally encouraged to give a maximal effort during the test. All participants were familiar with progressive maximal running tests as part of their usual fitness assessment program. The first ventilatory VT (VT_1_) was described as the point of transition from moderate to heavy intensity activity, and corresponds to the level of intensity at which the first disproportionate increment in ventilation (VE) to *V*O_2_ consumption occurs. The second disproportionate increase in ventilation is labelled the second VT (VT_2_), and represents the highest sustainable level of exercise intensity. As recommended [14], VT_1_ and VT_2_ were determined by two independent experienced technicians. *V*O_2peak_ was considered as the highest 15-s average achieved during the test [27] and was considered valid if two of the following criteria were attained [28]: (*i*) volitional exhaustion, (*ii*) heart rate within 10% of the age-specific estimated maximal heart rate [HRmax = 220 –age], (*iii*) a plateau in oxygen consumption despite increased exercise intensity (ΔVO_2_ between 2 consecutive work rates < 2.1 mL·kg^-1^·min^-1^); (*iv*) a maximal respiratory exchange ratio ≥ 1.10.

### Statistical analysis

Descriptive statistics (means and standard deviations) were calculated. Normality was checked with the Shapiro-Wilk test. Pearson correlations were calculated to examine the linearity of relationships among indicators of body size with *V*O_2peak_, VT_2_ and VT_1_ expressed in absolute terms (L·min^-1^). Allometric models were used to examine the relationship between body size descriptors and *V*O_2_ parameters (peak, VT_2_, and VT_1_):

$$y=a\;\times \;x^{k}\;\times \;\varepsilon$$ (2)

where “*y”* was the dependent variable of *V*O_2_ (peak, VT_2_ and VT_1_) and values of “*a”* and “*k”* were derived from linear regressions of the logarithmic regression transformations in the form of:

logy=loga+k×logx+logε (3)

where “*a”* was the scaling constant, and “*k”* was the scaling exponent for the specific body size descriptor (body mass or fat-free mass). Relationships among residuals and scaled variables were examined for each allometric model using Pearson correlations. The magnitude of correlations was interpreted as trivial (r < 0.1), small (0.1 < r < 0.3), moderate (0.3 < r < 0.5), large (0.5 < r < 0.7), very large (0.7 < r < 0.9), and nearly perfect (r > 0.9) [29].

CA, anthropometric variables and *V*O_2_ outputs expressed in absolute terms and as ratio standard and scaled values were compared between average and early maturing players using student t-tests for independent samples. The magnitude of the differences was assessed using standardized mean differences (Cohen’s *d* effect size) with thresholds of 0.20, 0.60, 1.20, 2.0 and 4.0 for small, moderate, large, very large and extremely large [30]. Statistical significance was set at 5%. Analyses were done with SPSS (SPSS version 17.0, Chicago, Illinois, USA) and GraphPad Prism (GraphPad Prism 5.0 Software Inc, San Diego, CA).

## Results

Descriptive statistics are summarized in Table 1. CAs ranged from 12.36 to 15.41 years (range 3.05 years), while SAs ranged from 11.92 to 17.21 years (range 5.29 years). All anthropometric variables and physiological parameters derived from the incremental maximal treadmill test were normally distributed.

**Table 1:** Descriptive statistics (n = 47) and normality of the distributions.

Variables		Descriptive statistics				Normality (Shapiro-Wilk)	
	unit				Standard deviation		
		Mean	SEM	(95% CI)		value	p
Chronological Age	years	14.05	0.12	(13.81 to 14.30)	0.83	0.956	0.08
Skeletal age	years	15.33	0.18	(14.95 to 15.70)	1.27	0.962	0.13
Training experience	years	3.42	0.20	(3.02 to 3.82)	1.34	0.951	0.05
Stature	cm	167.4	1.5	(164.5 to 170.4)	10.0	0.975	0.41
Body mass	kg	56.4	1.5	(53.3 to 59.5)	10.5	0.964	0.16
Fat mass	%	12.9	0.4	(12.0 to 13.7)	2.8	0.977	0.48
Fat mass	kg	7.4	0.4	(6.7 to 8.1)	2.5	0.969	0.24
Fat-free mass	kg	49.0	1.5	(46.5 to 51.6)	8.6	0.979	0.54
VT_1:_ speed	km·h^-1^	10.35	0.19	(9.98 to 10.72)	1.27	0.974	0.37
VT_1:_ VO_2_	L·min^-1^	2.56	0.07	(2.43 to 2.70)	0.45	0.979	0.57
VT_1:_ VCO_2_	L·min^-1^	2.23	0.06	(2.10 to 2.35)	0.41	0.986	0.83
VT_1:_ RER		0.87	0.00	(0.85 to 0.88)	0.05	0.953	0.06
VT_1:_ % VO_2_ peak	%	75.6	1.06	(73.5 to 77.6)	6.9	0.981	0.63
VT_1:_ Heart rate	Bpm	160	1.9	(156 to 163)	13	0.975	0.40
VT_2:_ speed	km·h^-1^	13.62	0.16	(13.29 to 13.95)	1.12	0.980	0.57
VT_2:_ VO_2_	L·min^-1^	3.11	0.08	(2.94 to 3.28)	0.57	0.977	0.49
VT_2:_ VCO_2_	L·min^-1^	3.09	0.08	(2.91 to 3.26)	0.58	0.966	0.19
VT_2:_ RER		0.99	0.00	(0.98 to 1.00)	0.05	0.980	0.59
VT_2:_ % VO_2_ peak	%	91.2	0.6	(90.0 to 92.3)	3.8	0.954	0.06
VT_2:_ Heart rate	bpm	185	1.5	(182 to 188)	10	0.982	0.66
Peak VO_2_: speed	km·h^-1^	15.95	0.16	(15.61 to 16.28)	1.13	0.968	0.22
Peak: RER		1.16	0.01	(1.14 to 1.18)	0.07	0.987	0.87
Peak: Heart rate	bpm	200	1.6	(197 to 204)	11	0.958	0.09
Peak VO_2_	L·min^-1^	3.42	0.10	(3.22 to 3.61)	0.66	0.978	0.50

Abbreviations: VT_1_, first ventilatory threshold; VT_2_, second ventilatory threshold; VO_2peak_, peak oxygen uptake; VCO_2_, carbon dioxide expired; RER, respiratory exchange ratio; bpm, beats per minute; SEM, standard error of the mean; 95% CI, 95% confidence interval.

Bivariate correlations between log transformed VT_1_ with log transformed stature, body mass, and estimated fat-free mass were moderately high: 0.76, 0.85, and 0.84, respectively (Table 2). Corresponding correlations for log transformed VT_2_ and *V*O_2peak_ were slightly higher: 0.83, 0.91 and 0.91 between VT_2_ with, respectively, stature, body mass, and fat-free mass; and 0.85, 0.92, and 0.92 between *V*O_2peak_ with, respectively, stature, body mass and fat-free mass.

**Table 2:** Allometric modelling of log transformed VT_1_, VT_2_ and *V*O_2peak_ using log transformed indicators of body size.

Yi	Xi	allometric models [log Yi = log *a* + *k* × log Xi + log e]						
		Coefficients				model summary		
		A	k-exponent			r	SEE	adjusted R^2^
			value	standard error	(95% CI)			
Log VT_1_	Log stature	-11.202	2.369	0.298	(1.768 to 2.970)	0.764	0.122	0.574
	Log body mass	-2.289	0.801	0.075	(0.649 to 0.952)	0.846	0.101	0.710
	Log fat-free mass	-2.341	0.843	0.083	(0.676 to 1.009)	0.835	0.103	0.691
Log VT_2_	Log stature	-12.589	2.678	0.269	(2.137 to 3.218)	0.830	0.110	0.682
	Log body mass	-2496	0.900	0.060	(0.780 to 1.020)	0.914	0.080	0.831
	Log fat-free mass	-2578	0.953	0.066	(0.821 to 1.085)	0.908	0.082	0.820
Log VO_2_peak	Log stature	-13.294	2.834	0.272	(2.287 to 3.380)	0.841	0.111	0.701
	Log body mass	-2.600	0.949	0.059	(0.831 to 1.068)	0.923	0.079	0.849
	Log fat-free mass	-2.681	1.004	0.066	(0.872 to 1.136)	0.916	0.082	0.836

Abbreviations: VT_1_, oxygen uptake at the first ventilatory threshold; VT_2_, oxygen uptake at the second ventilatory threshold; VO_2peak_, peak oxygen uptake.

The simple allometric models for VT_1_, VT_2_ and *V*O_2peak_ are summarized in Table 2. The 95%CI for the scaling exponents suggested linearity for all combinations, except between log-transformed scores of VT_1_ and body mass (beta exponent = 0.80). The coefficients were slightly higher than the respective scores using the original data (before logarithmic transformation). The absolute residuals of the allometric models (e.g., VT_1_ and body mass) were not significantly correlated with the log-transformed scores of body size variables. Visual inspection of the absolute residuals did not suggest heteroscedasticity, indicating that scaled variables were independent of the size descriptors.

**Fig 1:**
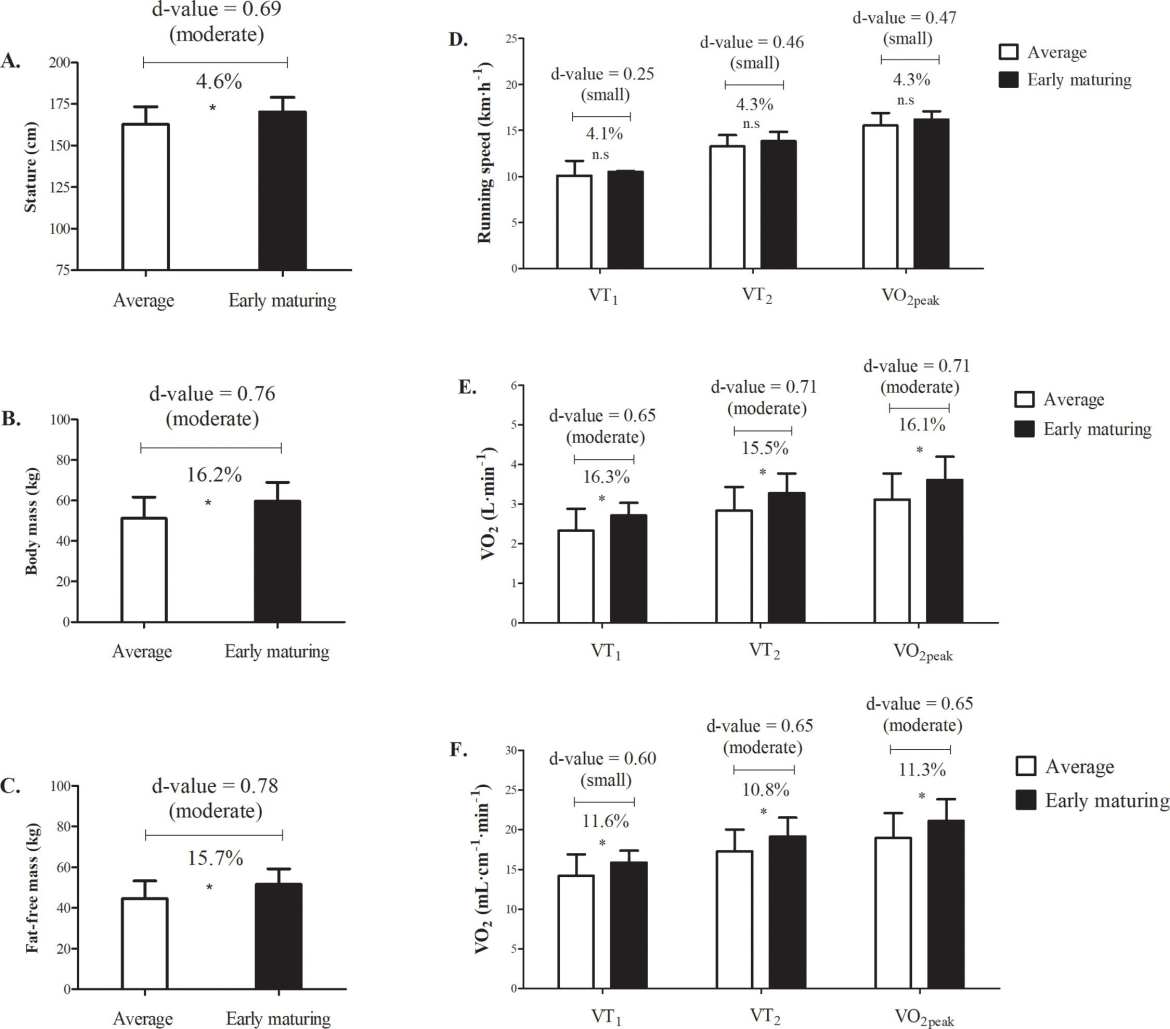
**Stature (panel A), body mass (panel B) and fat-free mass (panel C) in average maturing (white bars) and early maturing players (black bars). Mean differences between maturity groups [average maturing (white bars) and early maturing players (black bars)] regarding running speed (panel D), absolute peak oxygen uptake (*V*O**_**2peak**_**; panel E) and maximal metabolic conditions expressed by unit of stature (panel F) at each particular mark [first ventilatory threshold (VT**_**1**_**), second ventilatory threshold (VT**_**2**_**) and *V*O**_**2peak**_**].** * indicates difference between the groups (p<0.05).

Comparisons of average and early maturing soccer players are summarized in Table 3 and illustrated in Fig 1. Compared to average maturing players, early maturing players were, respectively, moderately (d = 0.69) taller [170.3±8.7 vs. 162.8±10.5 cm, t = -2.680, p = 0.01, Fig 1A] and heavier [59.6±9.4 vs. 51.3±10.4 kg, t = -2.828, p = 0.01, Fig 1B], and had a higher estimated fat-free mass [51.7±7.5 vs. 44.7±8.6 kg, t = -2.953, p = 0.01, Fig 1C]. Comparisons of absolute outputs for VT_1_ (2.71±0.32 vs. 2.33±0.55 L·min^-1^, t = -2.978, p = 0.01), VT_2_ (3.28±0.50 vs. 2.84±0.59 L·min^-1^, t = -2.724, p = 0.01) and *V*O_2peak_ (3.61±0.59 vs. 3.11±0.67 L·min^-1^, t = -2.692, p = 0.01) indicated moderately higher values (p<0.01) in early than in average maturing players, respectively (Fig 1E). In contrast, average and early maturing players, respectively, did not differ in running speeds at VT_1_ (10.09±1.61 vs. 10.51±0.10 km·h^-1^, t = -1.003, p = 0.33), VT_2_ (13.27±1.21 vs. 13.84±1.02 km·h^-1^, t = -1.753, p = 0.09) and *V*O_2peak_ (15.53±1.36 vs. 16.20±0.89 km·h^-1^, t = -1.844, p = 0.08); the effect sizes were small [Fig 1D].

**Table 3:** Descriptive statistics (mean ± standard deviation) for adolescent soccer players of contrasting skeletal maturity status and comparison of the ventilatory outputs between maturity groups expressed in absolute values and using ratio standard and scaled values.

Yi: Variable	Xi: descriptor	unit	Descriptive statistics		Comparisons		Effect size	
			Average maturing (n = 18)	Early maturing (n = 29)	t	p	d-value	(qualitative)
Chronological Age		years	14.01 ± 0.9	14.08 ± 0.8	-0.255	0.80	0.07	(trivial)
Skeletal Age		years	14.22 ± 1.00	16.02 ± 0.87	-6.495	0.00	1.72	(large)
Stature		cm	162.8 ± 10.5	170.3 ± 8.7	-2.680	0.01	0.69	(moderate)
Body Mass		kg	51.3 ± 10.4	59.6 ± 9.4	-2.828	0.01	0.76	(moderate)
Fat-Free Mass		kg	44.7 ± 8.6	51.7 ± 7.5	-2.953	0.01	0.78	(moderate)
VT_1_: Speed		km·h^-1^	10.09 ± 1.61	10.51 ± 0.10	-1.003	0.33	0.25	(small)
VT_1_: Absolute output		L·min^-1^	2.33 ± 0.55	2.71 ± 0.32	-2.978	0.01	0.65	(moderate)
VT_1_: Relative output	Body mass	mL·kg^-1^·min^-1^	45.58 ± 5.20	45.94 ± 5.00	-0.235	0.82	0.07	(trivial)
		mL·kg^-0.801^·min^-1^	99.37 ± 11.98	103.21 ± 9.12	-1.243	0.22	0.31	(small)
	Fat-free mass	mL·kg^-1^·min^-1^	52.23 ± 6.44	52.80 ± 5.26	-0.329	0.74	0.08	(trivial)
	Stature	mL·cm^-1^·min^-1^	14.23 ± 2.66	15.88 ± 1.51	-2.413	0.02	0.60	(small)
VT_2_: Speed	Speed	km·h^-1^	13.27 ± 1.21	13.84 ± 1.02	-1.753	0.09	0.46	(small)
VT_2_: Absolute output		L·min^-1^	2.84 ± 0.59	3.28 ± 0.50	-2.724	0.01	0.71	(moderate)
VT_2_: Relative output	Body mass	mL·kg^-1^·min^-1^	55.46 ± 4.15	55.22 ± 4.75	0.177	0.86	-0.06	(trivial)
	Fat-free mass	mL·kg^-1^·min^-1^	63.53 ± 4.83	63.50 ± 5.38	0.020	0.98	-0.01	(trivial)
	Stature	mL·cm^-1^·min^-1^	17.31 ± 2.74	19.18 ± 2.35	-2.488	0.02	0.65	(moderate)
Speed at peak VO2		km·h^-1^	15.53 ± 1.36	16.20 ± 0.89	-1.844	0.08	0.47	(small)
Absolute peak VO2		L·min^-1^	3.11 ± 0.67	3.61 ± 0.59	-2.692	0.01	0.71	(moderate)
Relative peak VO2	Body mass	mL·kg^-1^·min^-1^	60.72 ± 4.91	60.73 ± 4.87	-0.004	1.00	0.00	(trivial)
	Fat-free mass	mL·kg^-1^·min^-1^	69.52 ± 5.43	69.85 ± 5.86	-0.197	0.85	0.06	(trivial)
	Stature	mL·cm^-1^·min^-1^	18.97 ± 3.15	21.12 ± 2.74	-2.475	0.02	0.65	(moderate)

Abbreviations: VT_1_, first ventilatory threshold; VT_2_, second ventilatory threshold; VO_2peak_, peak oxygen uptake.

Given the significant body size differences between early and average maturing players, ratio standards (per unit body mass, fat-free mass and stature) were also used to compare VT_1_, VT_2_ and *V*O_2peak_. The scaled value for body mass as the size descriptor was also used for VT_1_ as this was the only scaling exponent that differed from linearity.

When tested using standard ratios, differences between maturity groups were non-significant and trivial for the three ventilatory variables (VT_1_, VT_2_, *V*O_2peak_). For the dependent variable that required an allometric model (VT_1_ using body mass as the size descriptor), the difference between maturity groups was small and not significant (d = 0.31, t = -1.243, p = 0.22). On the other hand, the results indicated significant differences between average and early maturing players for the three ventilatory outputs expressed per unit of stature (VT_1_: 14.23 ± 2.66 vs. 15.88 ± 1.51 mL·cm^-1^·min^-1^, t = -2.413, p = 0.02; VT_2_: 17.31 ± 2.74 vs. 19.18 ± 2.35 mL·cm^-1^·min^-1^, t = -2.488, p = 0.02; *V*O_2peak_: 18.97 ± 3.15 vs. 21.12 ± 2.74 mL·cm^-1^·min^-1^, t = -2.475, p = 0.02; respectively), although the magnitude effect was small for VT_1_ and moderate for VT_2_ and *V*O_2peak_ (Fig 1F).

**Fig 2:**
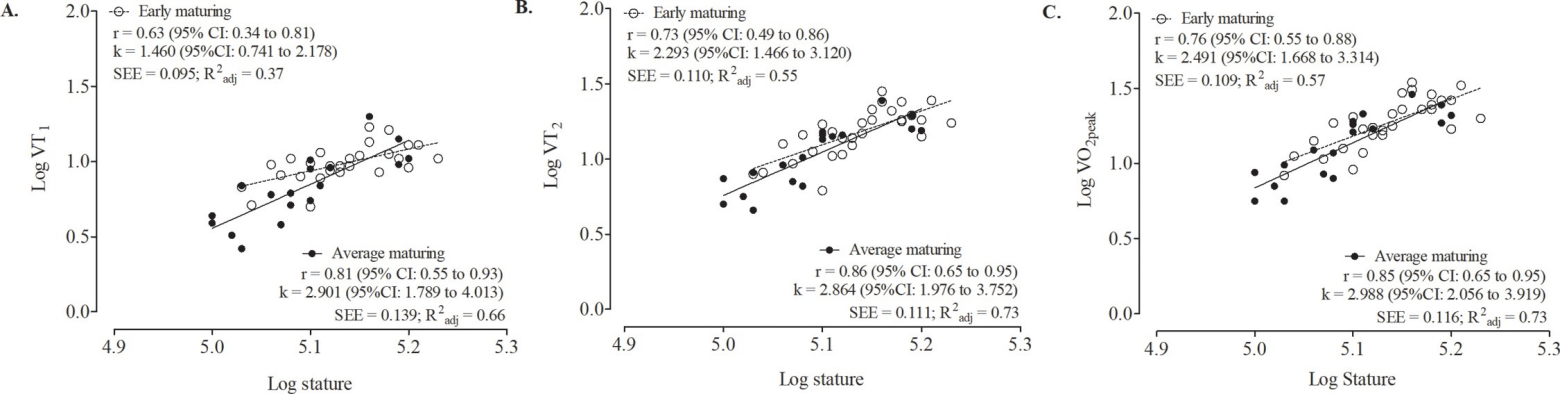
**Allometric scaling pattern of log transformed VO2 for different metabolic conditions: VT_1_ (panel A), VT_2_ (panel B) and *V*O_2peak_ (panel C)] using log transformed stature as the size descriptor in early and average maturing players.** Correlation coefficients (r), 95% confidence interval (95%CI), standard errors of estimate (SEE) and adjusted coefficients of determination (R^2^_adj_) are also presented.

Fig 2 illustrates the scaling pattern of ventilatory variables for average and early maturing players with stature as the size descriptor. For average maturing players, the stature-scaling *k*-exponents were 2.90 for VT_1_ (95%CI: 1.79 to 4.01; R^2^_adj_ = 0.66), 2.86 for VT_2_ (95%CI: 1.98 to 3.75; R^2^_adj_ = 0.73) and 2.99 for *V*O_2peak_ (95%CI: 2.06 to 3.92; R^2^_adj_ = 0.73). The corresponding stature-scaling *k*-exponents for early maturing players were 1.46 for VT_1_ (95%CI: 0.74 to 2.18; R^2^_adj_ = 0.37), 2.29 VT_2_ (95%CI: 1.47 to 3.12; R^2^_adj_ = 0.55) and 2.49 for *V*O_2peak_ (95%CI: 1.67 to 3.31; R^2^_adj_ = 0.57).

## Discussion

Interrelationships among skeletal maturity status, several indicators of body mass and stature on several oxygen ventilatory outputs were considered in adolescent male soccer players. Differences between players of contrasting maturity status favoured early maturing players who were taller and heavier (see Fig 1A and 1B) and also attained higher absolute values in ventilatory thresholds and *V*O_2peak_ compared to average maturing players (see Fig 1E). After normalizing for inter-individual variability in body mass and fat-free mass using a ratio standard and the scaled variable, the advantages of the early maturing players were attenuated. On the other hand, relative values of *V*O_2_ outputs normalized for stature indicated higher mean values for early compared to average players (see Fig 1F). The mean difference was more apparent for VT_2_ and *V*O_2peak_, which suggested a moderate difference in metabolic economy at the highest intensities associated with advanced skeletal maturity status. On the other hand, average maturing players were somewhat more efficient at identical running speeds.

Physical performances that require movement of body mass through space will benefit individuals who have greater stature-to-mass ratios [31]. Relative to U.S. age-specific reference data for males [32], mean stature and body mass of the sample of 47 Brazilian adolescent soccer players were higher than the respective reference medians (64th and 68th, for stature and body mass, respectively), consistent with observations for other samples of youth soccer players [16, 33, 34]. The advanced SA relative to CA of the present sample was also consistent with prior results for adolescent soccer players in Spain [35] and Portugal [33]. Absolute *V*O_2peak_ and VT_2_ of the players of the current sample were also similar to those observed in soccer players of the same CA and competitive level [14, 16]. As expected, absolute *V*O_2peak_ of the soccer players in this study was, on average, higher than observed in previous studies of physically active adolescents of similar age [27]. Note, however, the interpretation and comparison of VO_2_ values obtained from different ergometers, gas analysers and protocols (speed at baseline, length of each stage, slope, among other factors such as criteria for checking data quality) requires caution. Running on a treadmill, for example, utilizes a larger muscle mass than does pedalling on a cycle ergometer. As a result, the highest *V*O_2_ on a cycle ergometer is, on average, about 10% lower than on a treadmill [36]. The mean duration for the incremental ramp test was 14.6±1.9 min, resulting in a test duration close the recommended range of 8–12 min as previously suggested for healthy individuals [28, 37]. Furthermore, this protocol has successfully been used to elicit *V*O_2peak_ and to determine speed associated to VT and *V*O_2peak_ during treadmill running in young athletes [38].

Scaling coefficients using body mass as a size descriptor to normalize *V*O_2_ outputs during submaximal intensities (70–85% *V*O_2peak_) ranged 0.88–0.95 in both physically active and trained adolescents [14, 27]. In Portuguese adolescent soccer players, coefficients for log transformed *V*O_2peak_ and body mass decreased from 0.98 at 8–12 years to 0.86 at 13–15 years and to 0.51 at 16–18 years [16]. The relationship between log-transformed *V*O_2_ parameters and body size in the current study tended to fluctuate above and below the line of unity, suggesting a linear relationship between the variables. Thus, the same scaling coefficient may not be appropriate for all ages [39]. Second, the exponents 0.67 or 0.75, which are commonly recommended for partitioning the influence of body mass on *V*O_2peak_ [14, 18, 27] may not be valid. Third, allometric coefficients should be developed for each particular sample [40]. Moreover, among highly trained adolescent soccer players, the postulates underling the use power functions among tri-dimensional size descriptors and oxygen consumption may be potentially confounded by the effects of training including an increase in fat-free and muscle mass [16], a reduction in fat mass and changes in movement efficiency [41]. The selective retention of taller players at elite levels [25] may also influence the scaling exponents for stature.

Given the substantial variability in individual growth patterns in scaled *V*O_2peak_, derivation of a single scaling factor is problematic. Estimated lean soft tissue of the lower limbs combined with pubertal status (pubic hair stages 3 and 4) explained a significant proportion of inter-individual variability in *V*O_2peak_ (41%) in a sub-sample of soccer players 13 to 15 years [16]. Note, however, stages of pubic hair are discrete (the individual is either in stage or not in stage) and as such may not be adequate for use with statistical techniques, which assume continuous variables with a normal distribution. The allometric models in the present study explained a large proportion of the variance in *V*O_2peak_, 70% to 85%.

The current study did not include any players classified as late maturing. This was somewhat surprising as it contrasted the available SA literature for youth soccer players 12 to 15 years of age which commonly include late maturing players between 12 and 15 years of age, although numbers vary with method of SA assessment [19]. Nevertheless, numbers of late maturing players decline with increasing CA which suggests that the sport of soccer systematically excludes late maturing players in favour of average and early maturing players as adolescence progresses.

The scaling of oxygen ventilatory outputs varied between the maturity groups. Progressively higher size exponents (*k* = 1.46–2.49) were noted in early maturing players (*r* = 0.63–0.76; R^2^_adj_ = 0.37–0.57) as they progressed from sub-maximal exercise to maximal metabolic conditions but not in average maturing players (*k* = 2.86–2.99; *r* = 0.81–0.86; R^2^_adj_ = 0.66–0.73). Overall, the results suggested that ventilatory outputs were dependent to a large extent on the player’s physiological state [18] as expressed in his skeletal maturity status. Average maturing and smaller athletes, in contrast, exhibited greater capacity of the cardiovascular system and better running economy. Of potential relevance, the kinetic characteristics of running style in six early maturing and seven late maturing soccer players (based on SA) 12–16 years of age (mean 14.3 years) indicated longer strides relative to leg length at 8 km.h^-1^, 9.5 km∙h^-1^ and 11 km∙h^-1^ among late maturing youth players [42]. In addition, development of aerobic performance as assessed with the Yo-Yo Intermittent Recovery Test (level 1) in 162 elite soccer players 11–1 years was related to muscularity and by inference to motor coordination [43].

The current investigation has several strengths, for example, objective measures of aerobic function, and also highlights the need to consider inter-individual variation in biological maturity status (using an established and valid indicator) and body size in the evaluation of aerobic fitness in competitive adolescent soccer players. Such variation can potentially confound the evaluation of players. There is also a need to experimentally determine and verify allometric exponents for partitioning the influence of body size and maturity status on *V*O_2_ during both submaximal and maximal exercises. Given the trend for the selectivity of taller, earlier maturing adolescent players in soccer, their apparent disadvantage in running and metabolic economy should be noted. Coaches may perhaps implement motor-coordination drills into training programs at these ages.

Some methodological limitations should also be noted. Although SA is generally considered the best indicator of maturity status, obtaining radiographs is costly and its examination requires experienced assessors. Furthermore, the lack of late maturing players in the present sample did not permit comparisons among boys at the extremes of skeletal maturity status (late vs. early). The limitation of the relatively small sample size and the use of predicted body composition descriptors should also be noted.

## Conclusions

Skeletal maturity status and associated variation in overall body size affected VT_1_, VT_2_ and *V*O_2peak_ responses varied with maturity status in elite adolescent soccer players. Inter-individual variability in mass descriptors (body mass and fat-free mass) using a ratio standard and the scaled variable attenuated the apparent higher values of oxygen uptake of the early maturing players noted for absolute values. In contrast, scaling exponents for stature increased with increasing metabolic demands in early maturing players, but were stable across increasing metabolic demands in average maturing players. The observed scaling pattern of ventilatory outputs for body mass and stature may be related to the smaller body size and better running economy of average maturing athletes.

## Supporting information

S1 File Full dataset. (XLSX)
